# Intravitreal triamcinolone versus laser photocoagulation as a primary treatment for diabetic macular oedema - a comparative pilot study

**DOI:** 10.1186/1471-2415-11-36

**Published:** 2011-11-23

**Authors:** Mustapha Norlaili, Shaharuddin Bakiah, Embong Zunaina

**Affiliations:** 1 Department of Ophthalmology, School of Medical Sciences, Universiti Sains Malaysia, 16150 Kubang Kerian, Kelantan, Malaysia; 2 Advanced Medical and Dental Institute, Universiti Sains Malaysia, 13200 Kepala Batas, Pulau Pinang, Malaysia

## Abstract

**Background:**

Diabetic macular oedema is the leading causes of blindness. Laser photocoagulation reduces the risk of visual loss. However recurrences are common and despite laser treatment, patients with diabetic macular oedema experienced progressive loss of vision. Stabilization of the blood retinal barrier introduces a rationale for intravitreal triamcinolone treatment in diabetic macular oedema. This study is intended to compare the best corrected visual acuity (BCVA) and the macular oedema index (MEI) at 3 month of primary treatment for diabetic macular oedema between intravitreal triamcinolone acetonide (IVTA) and laser photocoagulation.

**Methods:**

This comparative pilot study consists of 40 diabetic patients with diabetic macular oedema. The patients were randomized into two groups using envelope technique sampling procedure. Treatment for diabetic macular oedema was based on the printed envelope technique selected for every patient. Twenty patients were assigned for IVTA group (one injection of IVTA) and another 20 patients for LASER group (one laser session). Main outcome measures were mean BCVA and mean MEI at three months post treatment. The MEI was quantified using Heidelberg Retinal Tomography II.

**Results:**

The mean difference for BCVA at baseline [IVTA: 0.935 (0.223), LASER: 0.795 (0.315)] and at three months post treatment [IVTA: 0.405 (0.224), LASER: 0.525 (0.289)] between IVTA and LASER group was not statistically significant (p = 0.113 and p = 0.151 respectively). The mean difference for MEI at baseline [IVTA: 2.539 (0.914), LASER: 2.139 (0.577)] and at three months post treatment [IVTA: 1.753 (0.614), LASER: 1.711 (0.472)] between IVTA and LASER group was also not statistically significant (p = 0.106 and p = 0.811 respectively).

**Conclusions:**

IVTA demonstrates good outcome comparable to laser photocoagulation as a primary treatment for diabetic macular oedema at three months post treatment.

**Trial Registration:**

ISRCTN05040192 (http://www.controlled-trial.com)

## Background

Diabetic macular oedema (DME) is the leading causes of blindness in an increasing number of patients with diabetes. Reduction of visual acuity in DME results from accumulation of fluid produced from a rupture of the blood-retinal barrier into the inner nuclear layer of the retina. The thickened macula can be visualized on slit lamp examination using 90 Dioptre or 78 Dioptre lens. The retinal thickness can be measured or quantified by Optical Coherent Tomography (OCT), Confocal laser scanning using Heidelberg Retina Tomography II (HRT II) or Retinal Thickness Analyzer.

Scanning laser tomography (SLT) in HRT II is a non-invasive technique which permits the objective, topographic measurement of the fundus. SLT employs confocal optics to attain a high resolution not only perpendicular to × and y axis but also along z axis (the optical axis). The distribution of reflected light intensity along the optical axis for a given pixel is described as the z-profile or confocal intensity profile. An oedema index can be derived for each pixel, which is sensitive to oedematous changes of the retina. A resultant map of these oedema indices gives a measure of the location and extent of retinal oedema. It should be noted that the macular oedema index (MEI) is not a measure of retinal thickness but reflects the changes of retinal thickness based on the retinal refractive index in the areas of oedema. The oedema index methodology has been validated in diabetic retinopathy but not in other disease states. Change of the oedema index has been shown to correlate with change of visual function, including logarithm of the minimum angle of resolution (log MAR) visual acuity, conventional automated static perimetry and short-wavelength automated perimetry, in patients undergoing grid laser treatment for clinically significant macular oedema [1].

Laser photocoagulation reduces the risk of visual loss in 60% of patients. However recurrences are common and despite laser treatment, 26% of patients with DME experienced progressive loss of vision [2]. Furthermore, 40% of treated eyes that had retinal oedema involving the centre of the macula at baseline still had oedema involving the centre at 12 months, as did 25% of treated eyes at 36 months [3]. The frequency of an unsatisfactory outcome following laser photocoagulation in some eyes with DME has prompted interest in other treatment modalities.

Intravitreal triamcinolone acetonide (IVTA) has been shown experimentally to reduce the breakdown of blood retinal barrier [4]. It down regulates the production of vascular endothelial growth factor; a known vascular permeability factor hence reduced the vascular permeability. Stabilization of the blood retinal barrier introduces a rationale for IVTA treatment in DME.

IVTA has proved to be effective in the treatment of DME from previous study. It constitutes a newer, less destructive treatment modality in the management of DME. Two previous studies of primary IVTA in DME [5,6] have shown improvement on visual acuity as well as central macular thickness. Massin et. al. compared the use of IVTA as an adjunctive therapy in DME eyes which failed laser treatment where it effectively reduced the macular thickening [7]. Jonas et. al. in 2003 reported in their prospective, interventional, clinical case series study, the visual acuity had significantly improved with IVTA [8].

This study is designed to compare the best corrected visual acuity (BCVA) and the macular oedema index (MEI) at 3 months of primary treatment for DME between IVTA and laser photocoagulation. Confocal laser scanning machine, HRT II is used to quantify the MEI pre and post treatment. To our knowledge, HRT II has never been used as an evaluation tool in comparative study to assess macular oedema in DME before and after treatment.

## Methods

### Subjects

A comparative pilot study was conducted from June 2007 to February 2008, at Hospital Universiti Sains Malaysia, Kelantan, Malaysia. The sample size was calculated using 'Power and Sample Size' software, version 2.1.31. Based on the research design and strategy, sample size was calculated using 'two proportions formula' model with 90% power of study. It was calculated based on improvement of visual acuity in IVTA, 81% [8] and 25% in laser photocoagulation group [9]. A total 40 patients (20 per arm) was required for this study.

Diabetic patients with newly diagnosed clinically as DME, and age more than 18 years old were included in this study. Patients with media opacity impairing intravitreal injection or laser photocoagulation procedure, DME with proliferative diabetic retinopathy still undergoing pan retinal photocoagulation, history of ocular surgery (eg. cataract operation) or Yag procedure with the risk of further aggravating the macular oedema, intra-ocular pressure > 25 mmHg or any established glaucoma patient, ocular or systemic infection, known steroid allergy or responder, history of systemic steroid within 4 months prior to randomization and HbA_1c _more than 10% were excluded from the study.

### Sampling Procedure

Envelope technique sampling procedure was conducted. A stack of opaque envelope was prepared with 20 envelopes containing a piece of paper with the word 'IVTA' and the remaining 20 envelopes stated 'LASER'. The envelope was drawn for each patient by a co-investigator. This was performed once the patient had agreed to be included in the study.

### Study Procedure

All patients underwent a complete ocular and systemic assessment once they consented for the study. The assessment was performed by the primary investigator before they were randomized into the two groups.

### 1. Pre-Treatment Parameters Measurements

#### 1.1 Visual acuity

Visual acuity of both eyes was tested with the standard retro illuminated Snellen chart. BCVA for each eye was recorded in logarithm of the minimum angle of resolution (log MAR) notations [10] and used as a baseline.

All patients underwent subjective refraction by one optometrist. This is important as any astigmatism of -1 Dioptre and more need to be corrected with astigmatism lens before proceeding with the HRT II for measurement of MEI.

#### 1.2. Fundus examination

Fundus examination was done using 78 Dioptre lens on slit lamp bio microscopy and binocular indirect ophthalmoscopy. DME was classified as mild, moderate and severe based on the International Clinical Diabetic Macular Oedema Disease Severity Scale [11].

#### 1.3 Macular oedema index

MEI analysis has been incorporated within the HRT II as the macular oedema mapping (MEM). The baseline MEM was taken using the HRT II. Patients were properly positioned in front of the HRT II system with their full correction of astigmatism if any. The focus was then adjusted to get a clear image of the macula formed on the monitor. Three sets of three consecutive images were captured each time. To ensure image quality and proper handling, all guidelines recommended by the manufacturer were followed.

The best image was chosen based on the quality and smallest standard deviation. One good quality scan of each eye was utilised in all analyses. A 0.5 mm diameter circle was drawn using the circle draw facility of the HRT II. The area was chosen based on the most oedematous area and the same area was marked for the follow up photograph at three months. Measurement of MEI was performed by a blinded trained medical technician. After the baseline measurement of MEI, all the patients were randomized using the envelope technique. The type of treatment selected would be performed the next day.

### 2. Treatment Procedure

#### 2.1 Laser photocoagulation

Patients were properly positioned on a stable chair with the chin rested on the slit lamp that was mounted with a laser wavelength, Carl Zeiss Visulas 532S laser system. Patients were given grid or focal laser depending on the type of the macular oedema. Topical anaesthetic, 5% proparacaine hydrochloride was instilled in the eye which needed to be lasered. The laser settings were 50 micron spot size, duration of 0.1 seconds and appropriate power started from 50 mW and stepped up till it burned the retina with light gray burn. The number of laser burn given was based on the severity of diabetic macular oedema (range: 20 - 200 laser burns and 500 μm away from the centre of the fovea). Only one session of laser (either focal or grid laser) was given to each patient in LASER group. The procedure was done by Investigator A (ophthalmologist). Patient was follow-up at 3 months post laser and no other treatment was given during that period.

#### 2.2 Intravitreal triamcinolone acetonide

Intravitreal injection of triamcinolone was carried out under sterile conditions in the operation room. Patient was admitted on a day care basis. Topical chloramphenicol four times a day was prescribed one day prior to procedure. The procedure was done under local anaesthesia using topical 5% proparacaine hydrochloride. The selected eye was properly cleaned and draped. An eye speculum was then applied; flush irrigation with 5 mls 5% povidone iodine was performed on the eye for one minute.

Triamcinolone acetonide in a single-use vial (40 mg/ml, 1 ml vial), was drawn into a 1-cc tuberculin syringe after cleansing the top of the bottle with an alcohol wipe. A separate 27 gauge needle was placed onto the syringe, which was then inverted to remove air bubbles. The excess triamcinolone was discarded till 0.1 ml (4 mg) remained in the syringe.

The site of injection was then identified, at 3.5 mm in pseudophakic and 4 mm in phakic eye to ensure against passage of the needle through the vitreous base. It was given at the inferotemporal region to avoid drug deposition in front of the visual axis. Triamcinolone acetonide of 4 mg in 0.1 mls was injected into the vitreous using a 27-gauge needle transconjunctivally. Using a single, purposeful continuous manoeuvre, the 4 mg triamcinolone acetonide was injected into the eye. The needle was removed simultaneously with the application of cotton tipped applicator over its entry site to prevent regurgitation of the injected material. Indirect ophthalmoscopy was performed to check for central retinal artery pulsation. The procedure was done by Investigator B (ophthalmologist). Topical chloramphenicol four times daily would be continued for one week. Only one injection of IVTA was given to each patient in IVTA group. Patient was follow-up at 3 months post IVTA and no other treatment was given during that period.

### 3. Post-Treatment Parameters Measurements

Patient was follow-up at 3 months post procedure. The similar step of visual acuity and MEI assessment as pre-treatment measurement was done. The outcome measures were mean BCVA and mean MEI.

### Statistical analysis

All the statistical method analysis was done with Statistical Package for Social Sciences (SPSS Inc) software, version 12.0. Normality was tested using Eye-balling (histogram pattern). Independent T-test, paired T-test and Chi square test were used to analyze the results where appropriate. The p value of < 0.05 is considered as statistically significant.

### Ways to minimise study error

The following steps were taken to reduce errors while conducting the study:-

i. Patients were selected strictly based on the inclusion and exclusion criteria.

ii. Randomization of patients.

iii. IVTA and laser photocoagulation were performed by experienced ophthalmologist who was masked to patient's identity. A standardised technique was used for both procedures.

iv. The measurement of MEI was performed by one identified and trained medical technician.

v. The primary investigator was masked to patient's identity and procedures when analyzing the MEI results (pre and post intervention) of all patients.

The study was approved by the Research and Ethical Committee, School of Medical Sciences, Universiti Sains Malaysia [Ref: USM/PPSP^®^/Ethics Com./2006(176.3(1)].

## Results

### Demographic data

A total of 40 patients were enrolled into this study. Twenty patients were assigned for IVTA group and another 20 patients for LASER group. Mean age, duration of Diabetes Mellitus (DM), and status of HbA_1c _of patients in IVTA and LASER group is shown in Table 1. There were 8 males (40%) and 12 females (60%) in the IVTA group while 11 males (55%) and 9 females (45%) in the LASER group. The severity of DME for both groups is shown in Table 2.

**Table 1:** Characteristic of patients in IVTA and LASER group at baseline

Variables	IVTA		LASER		(95% CI of mean difference)	*p value
	(n = 20)		(n = 20)			
	Mean	SD	Mean	SD		
Age (year)	58.65	7.26	56.85	6.40	(-2.58, 6.18)	0.411
Duration of DM (year)	8.40	3.98	8.35	4.98	(-2.83, 2.93)	0.972
HbA_1c _(mmols)	8.92	0.81	9.01	0.95	(-0.65, 0.48)	0.762

DM: Diabetes Mellitus, *Independent T-test, p < 0.05 significant

**Table 2:** Distributions of cases according to severity of DME

Severity of DME	IVTA		LASER		*p value
	(n = 20)		(n = 20)		
	n	%	n	%	
Mild	6	30	9	45	0.265
Moderate	8	40	9	45	
Severe	6	30	2	10	

DME: Diabetic macular oedema, *Chi square test, p < 0.05 significant

### Comparison of BCVA and MEI

The comparison of mean BCVA and MEI in both groups at baseline and at three months post treatment is shown in Table 3. The mean difference for BCVA and MEI within the group at baseline and at three months post treatment was statistically significant (p < 0.01). The comparison of mean BCVA and MEI between IVTA and LASER groups at baseline and three months post treatment is shown in Table 4. The mean difference for BCVA at baseline and at three months post treatment between IVTA and LASER was not statistically significant (p = 0.113 and p = 0.151 respectively). Similarly, the mean difference for MEI at baseline and at three months post treatment between IVTA and LASER group was also not statistically significant (p = 0.106 and p = 0.811 respectively).

**Table 3:** Comparison of best corrected visual acuity and macular oedema index within the group at baseline and at three months post treatment

	At baseline		At 3 months post treatment		(95% CI of mean difference)	*p value
	Mean	SD	Mean	SD		
Best Corrected Visual Acuity						
IVTA	0.935	0.223	0.405	0.224	(0.430, 0.629)	p < 0.01
LASER	0.795	0.315	0.525	0.289	(0.162, 0.377)	p < 0.01
Macular Oedema Index						
IVTA	2.539	0.914	1.753	0.614	(0.549, 1.022	p < 0.01
LASER	2.139	0.577	1.711	0.472	(0.252, 0.604)	p < 0.01

*Paired t-test, p < 0.05 significant

**Table 4:** Comparison of best corrected visual acuity and macular oedema index between IVTA and LASER groups at baseline and at three months post treatment

	IVTA (n = 20)		LASER (n = 20)		(95% CI of mean difference)	*p value
	Mean	SD	Mean	SD		
Best Corrected Visual Acuity						
At baseline	0.935	0.223	0.795	0.315	(-0.349, 0.315)	0.113
At 3 months post treatment	0.405	0.224	0.525	0.289	(-2.857, 0.457)	0.151

Macular Oedema Index						
At baseline	2.539	0.914	2.139	0.577	(-0.089, 0.889)	0.106
At 3 months post treatment	1.753	0.614	1.711	0.472	(-0.315, 0.400)	0.811

*Independent T test, p < 0.05 significant

## Discussion

We conducted this comparative pilot study to assess whether there was a significant difference between IVTA and laser photocoagulation with a single treatment as primary treatment of DME at three months by evaluating the BCVA and MEI. We used HRT II to evaluate the DME. We did not perform OCT to quantify the DME. MEM of HRT II showed very good agreement with fundus biomicroscopy in diabetic maculopathy [1].

In this study, the duration of DM in both groups were comparable (p = 0.972). Mean diabetic controlled as being shown by the HbA_1c _results were almost the same in each group, 8.92 (0.81) in IVTA and 9.01 (0.95) in LASER group. The HbA_1c _results showed moderate controlled of DM among our study samples. Our patients had poor control of DM compared to study by Batioglu and colleagues where the HbA_1c _was 4% to 6% [12].

In our study, we treat the patient either IVTA or laser for DME and review the mean BCVA and MEI at three months post procedure. Three months follow-up was chosen because only a single treatment was given. The requirement of re-treatment if needed will be given after three months. The mean BCVA in IVTA group at three months was 0.405 (0.224) and 0.525 (0.289) in LASER group. The mean difference at three months was not statistically significant (p = 0.151) which meant that neither IVTA nor laser were superior to each other as a primary treatment of DME at 3 months of treatment. Our result showed a comparable outcome with study done by Lam et al [13].

The significant improvement of BCVA in the IVTA group (p < 0.01) in our study was similar to the studies reported by few published data [1,14]. Our result also showed significant improvement of BCVA post laser therapy at three months (p < 0.01). However a study done by Lee et al [15] showed no significant improvement of BCVA at three months after laser treatment.

The mean MEI at three months in IVTA group was 1.753 (0.614) and 1.711 (0.472) in the LASER group. The mean difference of both groups was not statistically significant (p = 0.811). Both modalities demonstrated comparable outcome of reduction of MEI at three months. There was no published data on study using HRT II as an objective evaluation for DME post IVTA or laser treatments. Hence we could only compare our study with study using OCT measurement. Lam et al again reported comparable outcome of central macular thickness of IVTA and laser treatment at three months which was similar to our result [13].

The significant improvement of mean MEI at three months in the IVTA group in our study (p < 0.01) was comparable to previous studies using OCT evaluation [1,5,14,16-18]. We also found that the mean MEI in the LASER group also showed significant improvement at three months (p < 0.01). However, Lee et al [15] reported that there was no significant improvement of central macular thickness at 3 months after laser treatment. They found that for DME patients, the combination treatment (laser and IVTA) had a better therapeutic effect than the laser alone for improving BCVA and central macular thickness at the early follow-up time periods [15].

Limitation of this present study is our number of patients was relatively small and a bigger sample size would give a better and reliable result. Another limitation of this study was a short duration of follow up. A longer period of follow up, at least over 12 months would give more value especially to arrive a treatment recommendation and able to assess the side effect of Triamcinolone. The analysis of macular oedema may be improved by using alternative instrument like OCT to support the HRT II findings.

## Conclusions

Both IVTA and laser photocoagulation showed good comparable outcomes in term of BCVA and MEI at three months post treatment as primary treatment for DME.

## Competing interests

The authors declare that they have no competing interests.

## Authors' contributions

MN designed the study protocol, data collection and analysis. SB designed the study protocol and data analysis. EZ designed the study protocol and management of the study. All authors read and approved the final manuscript.

## Pre-publication history

The pre-publication history for this paper can be accessed here:

http://www.biomedcentral.com/1471-2415/11/36/prepub

## References

[B1] Kisilevsky M Hudson C Flanagan JG Nrusimhadevara RK Guan K Wong T Mandelcorn M Lam WC Devenyi RG Agreement of the Heidelberg Retina Tomograph II macula edema module with fundus biomicroscopy in diabetic maculopathy Arch Ophthalmol 2006 124 3 337 342 10.1001/archopht.124.3.337 16534053

[B2] Sutter FK Simpson JM Gillies MC Intravitreal triamcinolone for diabetic macular edema that persists after laser treatment: three-month efficacy and safety results of a prospective, randomized, double-masked, placebo-controlled clinical trial Ophthalmology 2004 111 11 2044 2049 10.1016/j.ophtha.2004.05.025 15522370

[B3] Ip MS Intravitreal injection of triamcinolone: an emerging treatment for diabetic macular edema Diabetes Care 2004 27 7 1794 1797 10.2337/diacare.27.7.1794 15220269

[B4] Wilson CA Berkowitz BA Sato Y Ando N Handa JT de Juan E Jr Treatment with intravitreal steroid reduces blood-retinal barrier breakdown due to retinal photocoagulation Arch Ophthalmol 1992 110 8 1155 1159 10.1001/archopht.1992.01080200135041 1497531

[B5] Ozkiris A Evereklioglu C Erkili K Tamelik N Mirza E Intravitreal triamcinolone acetonide injection as primary treatment for diabetic macular edema Eur J Ophthalmol 2004 14 6 543 549 28221662 10.5301/EJO.2008.3464

[B6] Karacorlu M Ozdemir H Karacorlu S Alacali N Mudun B Burumcek E Intravitreal triamcinolone as a primary therapy in diabetic macular oedema Eye 2005 19 4 382 386 10.1038/sj.eye.6701512 15309024

[B7] Massin P Audren F Haouchine B Erginay A Bergmann JF Benosman R Caulin C Gaudric A Intravitreal triamcinolone acetonide for diabetic diffuse macular edema: preliminary results of a prospective controlled trial Ophthalmology 2004 111 2 218 245 10.1016/j.ophtha.2003.05.037 15019365

[B8] Jonas JB Kreissig I Sofker A Degenring RF Intravitreal injection of triamcinolone for diffuse diabetic macular edema Arch Ophthalmol 2003 121 1 57 61 12523885

[B9] Yanyali A Nohutcu AF Horozoglu F Celik E Modified grid laser photocoagulation versus pars plana vitrectomy with internal limiting membrane removal in diabetic macular edema Am J Ophthalmol 2005 139 5 795 801 10.1016/j.ajo.2004.12.017 15860282

[B10] Dehghan MH Ahmadieh H Ramezani A Entezari M Anisian A A randomized, placebo-controlled clinical trial of intravitreal triamcinolone for refractory diabetic macular edema Int Ophthalmol 2008 28 1 7 17 10.1007/s10792-007-9097-y 17589809

[B11] Wilkinson CP Ferris FL III Klein RE Lee PP Agardh CD Davis M Dills D Kampik A Pararajasegaram R Verdaguer JT Global Diabetic Retinopathy Project Group Proposed international clinical diabetic retinopathy and diabetic macular edema disease severity scales Ophthalmology 2003 110 9 1677 1682 10.1016/S0161-6420(03)00475-5 13129861

[B12] Batioglu F Ozmert E Parmak N Celik S Two-year results of intravitreal triamcinolone acetonide injection for the treatment of diabetic macular edema Int Ophthalmol 2007 27 5 299 306 10.1007/s10792-007-9072-7 17453151

[B13] Lam DSC Chan CKM Tang EWH Li KKW Fan DSP Chan WM Intravitreal triamcinolone for diabetic macular oedema in Chinese patients: six-month prospective longitudinal pilot study Clin Experiment Ophthalmol 2004 32 6 569 572 10.1111/j.1442-9071.2004.00903.x 15575825

[B14] Larson J Zhu M Sutter F Gillies MC Relation between reduction of foveal thickness and visual acuity in diabetic macular edema treated with intravitreal triamcinolone Am J Ophthalmol 2005 139 5 802 806 10.1016/j.ajo.2004.12.054 15860283

[B15] Lee HY Lee SY Park JS Comparison of photocoagulation with combined intravitreal triamcinolone for diabetic macular edema Korean J Ophthalmol 2009 23 3 153 158 10.3341/kjo.2009.23.3.153 19794940 PMC2739972

[B16] Ozdemir H Karacorlu M Karacorlu SA Regression of serous macular detachment after intravitreal triamcinolone acetonide in patients with diabetic macular edema Am J Ophthalmol 2005 140 2 251.e1 251.e6 10.1016/j.ajo.2005.03.004 15992756

[B17] Gibran SK Cullinane A Jungkim S Cleary PE Intravitreal triamcinolone for diffuse diabetic macular oedema Eye 2006 20 6 720 724 10.1038/sj.eye.6701992 16021193

[B18] Lam DSC Chan CKM Mohamed S Lai TYY Lee VYM Liu DTL Li KKW Li PSH Shanmugam MP Intravitreal triamcinolone plus sequential grid laser versus triamcinolone or laser alone for treating diabetic macular edema: six-month outcomes Ophthalmology 2007 114 12 2162 2167 10.1016/j.ophtha.2007.02.006 17459479

